# Results of a National Survey of Program Directors’ Perspectives on a Pass/Fail US Medical Licensing Examination Step 1

**DOI:** 10.1001/jamanetworkopen.2022.19212

**Published:** 2022-06-28

**Authors:** Om U. Patel, William K. Haynes, Kaitlin G. Burge, Ishant S. Yadav, Todd Peterson, Andres Camino, Nicholas J. Van Wagoner

**Affiliations:** 1 University of Alabama at Birmingham School of Medicine, Birmingham; 2 Department of Emergency Medicine, University of Alabama at Birmingham, Birmingham; 3 Division of Infectious Diseases, Department of Medicine, University of Alabama at Birmingham, Birmingham

## Abstract

This survey study of medical residency program directors assesses differences in the relative importance of the US Medical Licensing Examination (USMLE) Step 1 in resident selection following its transition to pass/fail evaluation.

## Introduction

In January 2022, Step 1 of the US Medical Licensing Examination (USMLE) began reporting as pass/fail.^1,2^ How this change might affect resident selection and medical student advising remains unclear. This survey study assesses opinions of allopathic residency program directors (PDs) representing 25 specialties regarding USMLE examinations’ current ability to project clinical and specialty board examination performance, the relative importance of USMLE Step 1 in the current selection process for residents, and how the change to pass/fail will affect the relative importance of other factors in residency selection. While understanding program directors’ perceptions in aggregate has value, we hypothesized that differences exist by type of specialty. Analysis was performed comparing perceptions of procedural with nonprocedural specialty PDs.

## Methods

This survey study was approved by the institutional review board of the University of Alabama at Birmingham. All PDs provided informed consent when they agreed to participate in the survey. This study is reported following the American Association for Public Opinion Research (AAPOR) reporting guideline.

PD contact information for general surgery, vascular surgery, cardiothoracic surgery, orthopedic surgery, otolaryngology, plastic surgery, neurosurgery, internal medicine, neurology, psychiatry, pediatrics, family medicine, physical medicine and rehabilitation, urology, ophthalmology, dermatology, interventional radiology, radiation oncology, obstetrics and gynecology, diagnostic radiology, emergency medicine, pathology, internal medicine and pediatrics, child neurology, and anesthesiology was collected using the FREIDA (Fellowship and Residency Electronic Interactive Database Access) database. Contact information was available for 4232 PDs (representing 4926 total programs). In this survey study, PDs were asked to complete an anonymous online survey distributed via email weekly for 6 consecutive weeks. PDs were asked about residency selection criteria and the change of Step 1 to pass/fail (eAppendix in the Supplement). Responses were analyzed for all specialties and for subsets of procedural (general surgery, vascular surgery, cardiothoracic surgery, orthopedic surgery, otolaryngology, plastic surgery, and neurosurgery) and nonprocedural (internal medicine, neurology, psychiatry, pediatrics, family medicine, physical medicine, and rehabilitation) specialties. Groups were based on categorization established by Stanford Medicine.^3^ Specialties considered a mix of procedural and nonprocedural and those that are not a part of the National Residency Match Program were excluded. Multiple choice questions were analyzed using χ^2^ tests in RStudio using tidyverse and knitr packages. Nonprocedural specialties were set as the referent. Ranking questions were analyzed in MATLAB version 9.8 (MathWorks) using 2-tailed paired *t* tests comparing relative ranks of each variable before and after implementation, with α = .001. Data were analyzed from February 1 to April 1, 2022.

## Results

A total of 1029 PDs (24.3%) completed some portion of the survey (multiple choice questions, 1029 PDs [24.3%]; relative rankings, 773 PDs [17.7%]). Overall, 53.5% of PDs agreed that Step 1 predicted ability to pass specialty board examinations, and procedural specialty PDs were more likely to agree (70.3% vs 38.1%; *P* < .001); 50.8% of PDs agreed that Step 2 predicted ability to pass specialty board examinations, and there was no difference between subgroups (Table 1). Only relatively few PDs answered that Step 1 or Step 2 predicted clinical performance, and there were no differences between subgroups (Table 1). With Step 1 moving to pass/fail, 59.1% of PDs agreed that schools should share clerkship National Board of Medical Examiners shelf examination scores, and procedural specialties were more likely to agree (70.8% vs 52.0%; *P* < .001). While there were differences in the value placed on residency applicant characteristics by procedural and nonprocedural specialty PDs, there was little change in rank order of characteristics before and after the transition of Step 1 to pass/fail (Table 2). Step 2 clinical knowledge score had the largest change in rank overall, and this change was more prominent in procedural specialties (Table 2).

**Table 1.** Residency Program Directors’ Perspectives on Pass/Fail Scoring of the USMLE Step 1 Examination

Prompt	Program directors, % (99.9% CI)			*P* value
	Overall (N = 1029)	Procedural (n = 195)	Nonprocedural (n = 311)	
**Do you believe that USMLE Step 1 scores…**				
Adequately predict a resident’s ability to pass your specialty’s board exams?				
Yes	53.5 (48.4-58.6)^a^	70.3 (58.4-79.9)^a^^,^^b^	38.1 (30.0-47.0)	<.001
Neutral	26.4 (22.1-31.2)	19.5 (11.7-30.6)	32.2 (24.5-40.9)	
No	20.0(16.2-24.5)	10.3(4.9-19.9)	29.7 (22.2-38.3)	
Accurately predict a resident’s ability to perform clinically in your specialty?				
Yes	19.2 (15.4-23.6)	29.7 (20.1-41.6)	14.4 (9.2-21.8)	.60
Neutral	26.5 (22.2-31.3)	33.3 (23.3-45.1)	20.6 (14.3-28.7)	
No	54.3 (49.1-59.4)^a^	36.9 (26.3-49.0)	65.0 (56.2-72.9)^a^	
**Do you believe that USMLE Step 2 CK scores…**				
Adequately predict a resident’s ability to pass your specialty’s board exams?				
Yes	50.8 (45.7-56.0)^a^	46.7 (35.2-58.5)	56.5 (47.6-65.0)^a^	.21
Neutral	31.1 (26.5-36.0)	35.9 (25.4-47.9)	28.2 (21.0-36.8)	
No	18.1 (14.5-22.4)	17.4 (10.1-28.3)	15.3 (9.9-22.7)	
Accurately predict a resident’s ability to perform clinically in your specialty?				
Yes	30.4 (25.9-35.3)	35.9 (25.4-47.9)	33.3 (25.7-42.0)	.14
Neutral	36.5 (31.7-41.6)	41.0 (30.0-53.0)	29.9 (22.5-38.6)	
No	33.1 (28.4-38.1)	23.1 (14.5-34.5)	36.7 (28.7-45.6)	
**After USMLE step 1 becomes pass/fail…**				
Should medical schools share clerkship NBME shelf examination scores with residency programs?				
Yes	59.1 (54.0-64.1)^a^	70.8 (58.9-80.4)^a^^,^^b^	51.4 (42.6-60.1)^a^	<.001
Neutral	29.1 (24.7-34.0)	24.1 (15.4-35.68)	29.7 (22.2-38.3)	
No	11.7 (8.8-15.5)	5.1 (1.8-13.4)	18.9 (12.9-26.8)	
Will a student’s medical school rank be considered more?				
Yes^a^	60.3 (55.2-65.2)	66.2 (54.2-76.4)	52.0 (43.2-60.7)	.19
Neutral	21.1 (17.2-25.6)	20.5 (12.5-31.7)	23.7 (17.0-32)	
No	18.6 (14.9-22.9)	13.3 (7.0-23.6)	24.3 (17.5-32.6)	

Abbreviations: CK, clinical knowledge; NBME, National Board of Medical Examiners; USMLE, US Medical Licensing Examination.

^a^ Statistically significant (*P* < .001) plurality of responses: procedural included general surgery, vascular surgery (integrated), cardiothoracic surgery (integrated), orthopedic surgery, otolaryngology, plastic surgery (integrated), and neurosurgery; nonprocedural included internal medicine, neurology, psychiatry, pediatrics, family medicine, and physical medicine and rehabilitation.

^b^ Significant (*P* < .001) difference in response between procedural and nonprocedural groups based off χ^2^ tests, with nonprocedural as the reference group.

**Table 2.** Relative Rankings by Program Directors of Various Factors Before and After Implementation of Step 1 Pass/Fail

Variable	Overall (N = 1029)			Procedural (n = 195)^a^			Nonprocedural (n = 311)^a^		
	Ranking before/after	Rank (99.9% CI)		Ranking before/after	Rank (99.9% CI)		Ranking before/after	Rank (99.9% CI)	
		Before	After		Before	After		Before	After
Clerkship grades	1/1^b^	5.03 (4.65-5.42)^b^	4.57 (4.21-4.93)^b^	3/2^b^	5.58 (4.52-6.64)^b^	5.09 (4.05-6.14)^b^	1/1	4.93 (4.28-5.58)	4.60 (3.99-5.20)
Dean’s letter	6/5^b^	7.76 (7.23-8.29)^b^	7.14 (6.65-7.63)^b^	11/9^b^	9.91 (8.65- 11.18)^b^	9.19 (8.01- 10.37)^b^	2/2	6.24 (5.43-7.06)	5.97 (5.19-6.75)
Class rank	4/3^b^	6.38 (5.90-6.85)^b^	5.82 (5.38-6.28)^b^	4/4^b^	6.86 (5.67-8.05)^b^	5.93 (4.84- 7.02)^b^	3/3	6.48 (5.69-7.27)	6.10 (5.33-6.87)
Letters of recommendation	3/2	5.61 (5.13-6.09)	5.48 (5.02-5.94)	2/1	4.08 (3.09-5.08)	4.34 (3.30-5.37)	5/5	6.70 (5.87-7.53)	6.41 (5.63-7.20)
Step 2 CK score	5/4^b^	7.50 (6.90-8.10)^b^	6.15 (5.56-6.75)^b^	5/3^b^	7.53 (5.94-9.13)^b^	5.39 (3.84-6.93)^b^	4/4^b^	6.69 (5.73-7.66)^b^	6.10 (5.12-7.08)^b^
Personal statement	7/6^b^	7.89 (7.38-8.40)^b^	7.50 (7.03-7.97)^b^	9/8^b^	8.91 (7.68- 10.13)^b^	8.28 (7.08-9.47)^b^	6/6	6.72 (5.89-7.56)	6.51 (5.74-7.28)
Step 1 score	2/NA^b^	5.44 (4.91-5.96)^b^	NA^b^	1/NA	3.72 (2.64-4.80)	NA	7/NA	6.90 (5.94-7.85)	NA
Involvement and leadership	8/8	8.31 (7.78-8.84)	8.20 (7.68-8.71)	10/10	9.72 (8.41-11.03)	9.61 (8.34- 10.89)	8/7	7.15 (6.30-8.00)	7.17 (6.30-8.04)
Volunteering	11/11^b^	9.92 (9.46-10.38)^b^	9.07 (8.64-9.50)^b^	15/13^b^	11.27 (10.18- 12.36)^b^	10.20 (9.14-11.26)^b^	9/8^b^	8.80 (8.03-9.57)^b^	8.04 (7.34-8.74)^b^
Gold humanism honor society membership	10/9^b^	9.81 (9.32-10.32)^b^	9.04 (8.57-9.50)^b^	12/11^b^	10.97 (9.87-12.08)^b^	9.93 (8.81-11.06)^b^	10/9^b^	8.82 (7.96-9.68)^b^	8.32 (7.53-9.11)^b^
Alpha omega alpha membership	9/7^b^	8.58 (8.03-9.13)^b^	7.90 (7.39-8.41)^b^	6/6	7.71 (6.46-8.95)	7.38 (6.22-8.54)	11/10^b^	9.28 (8.34-10.21)^b^	8.59 (7.71-9.46)^b^
Preclinical grades	14/13^b^	10.93 (10.45-11.42)^b^	9.78 (9.31-10.24)^b^	14/12^b^	11.23 (9.96-12.51)^b^	10.02 (8.83-11.20)^b^	12/11^b^	10.68 (9.85-11.51)^b^	9.61 (8.81-10.42)^b^
Abstracts, presentations, and publications	12/10^b^	9.99 (9.53-10.45)^b^	9.06 (8.61-9.51)^b^	7/5^b^	7.84 (6.75-8.92)^b^	6.84 (5.82-7.85)^b^	13/13^b^	11.56 (10.86-12.26)^b^	10.57 (9.88-11.26)^b^
Mean No. of research experiences in specialty	13/12^b^	10.30 (9.83-10.78)^b^	9.24 (8.77-9.72)^b^	8/7^b^	8.42 (7.28-9.56)^b^	7.48 (6.36-8.61)^b^	14/12^b^	11.76 (11.01-12.51)^b^	10.41 (9.63-11.18)^b^
Away rotation in specialty	15/14^b^	11.50 (10.95-12.06)^b^	10.76 (10.25-11.27)^b^	13/14^b^	11.14 (9.69-12.60)^b^	10.41 (9.06-11.75)^b^	15/14^b^	12.48 (11.67-13.29)^b^	11.48 (10.71-12.24)^b^
Graduate degree	16/15^b^	13.65 (13.28-14.03)^b^	12.85 (12.49-13.20)^b^	16/15^b^	13.79 (12.81-14.77)^b^	12.84 (11.85-13.82)^b^	16/15^b^	13.41 (12.78-14.04)^b^	12.63 (12.04-13.23)^b^
Graduated top 40 NIH-funded school	17/16^b^	14.38 (13.90-14.86)^b^	13.42 (12.93-13.91)^b^	17/16^b^	14.29 (13.02-15.56)^b^	13.09 (11.69-14.49)^b^	17/16^b^	14.41 (13.61-15.21)^b^	13.50 (12.70-14.31)^b^

Abbreviations: CK, clinical knowledge; NA, not applicable; NIH, National Institutes of Health.

^a^ Procedural includes general surgery, vascular surgery (integrated), cardiothoracic surgery (integrated), orthopedic surgery, otolaryngology, plastic surgery (integrated), and neurosurgery. Nonprocedural includes internal medicine, neurology, psychiatry, pediatrics, family medicine, and physical medicine and rehabilitation.

^b^ Statistically significant (*P* < .001) plurality of responses by nonoverlapping 99.9% CIs within group.

## Discussion

Studies show that PDs in aggregate want more objective measures of applicant performance to guide the resident selection process.^4,5^ While our study confirms this, our results also demonstrate differences between PDs in procedural and nonprocedural specialties regarding the value they place on objective measures before and after the transition of Step 1 to pass/fail, with procedural specialties prioritizing USMLE examinations. The relative importance of academic performance characteristics, personal characteristics, and knowledge of applicant in resident selection was projected to remain similar following the transition of Step 1 to pass/fail.

Specialty-specific analyses are needed to understand the importance of these factors following the transition of Step 1 to pass/fail and guide medical students applying for residency. Our response rate was comparable with the National Resident Matching Program PD survey, but may not provide a comprehensive assessment of all PDs.^6^ Despite this limitation, this study provides insight into PDs’ perceptions about the transition to a pass/fail Step 1 as part of the resident selection process.

## Notes

Supplement. **eAppendix.** Survey Questions
