# Supplementary material for: Results of a National Survey of Program Directors’ Perspectives on a Pass/Fail US Medical Licensing Examination Step 1
Source: JAMA Netw Open. 2022 Jun 28;5(6):e2219212. doi: 10.1001/jamanetworkopen.2022.19212 (PMC9240899; doi:10.1001/jamanetworkopen.2022.19212)
Supplement: Supplement. — eAppendix. Survey Questions [file jamanetwopen-e2219212-s001.pdf]

Supplemental Online Content

Patel OU, Haynes WK, Burge KG, et al. Results of a national survey of program directors' perspectives on a pass/fail US Medical Licensing Examination Step 1. *JAMA Netw Open.* 2022;5(6):e2219212. doi:10.1001/jamanetworkopen.2022.19212

eAppendix. Survey Questions

This supplemental material has been provided by the authors to give readers additional information about their work.

© 2022 Patel OU et al. *JAMA Network Open.*

eAppendix. Survey Questions

Q0 Please indicate your position from the choices below.

- ○ Program Director (1)
- ○ Associate Program Director (2)
- ○ Assistant Program Director (4)

Q1 Please choose your specialty from the dropdown menu below.

▼ Internal Medicine (1) ... Child Neurology (25)

Q2 Are you a top 15 NIH funded program?

- ○ Yes (1)
- ○ No (2)
- ○ Don't Know (3)

Q3 How many applications do you receive per year (estimate)?

____

Q4 How many open spots do you have per year?

____

Q5 Are you an academic, academically-affiliated, or a community-based program?

- ○ Academic (1)
- ○ Academic-affiliated (2)
- ○ Community-Based (3)

© 2022 Patel OU et al. *JAMA Network Open.*

Q6 After *USMLE STEP 1* becomes pass/fail, should medical schools share clerkship NBME shelf exam scores with residency programs?

- ◯ Yes (1)
- ◯ Neutral (2)
- ◯ No (3)

Q7 Do you believe that *USMLE STEP 1* scores adequately predict a resident's ability to pass your specialty's board exams?

- ◯ Yes (1)
- ◯ Neutral (2)
- ◯ No (3)

Q8 Do you believe that *USMLE STEP 2CK* scores adequately predict a resident's ability to pass your specialty's board exams?

- ◯ Yes (1)
- ◯ Neutral (2)
- ◯ No (3)

Q9 Do you believe that *USMLE STEP 1* scores accurately predict a resident's ability to perform clinically in your specialty?

- ◯ Yes (1)
- ◯ Neutral (2)
- ◯ No (3)

© 2022 Patel OU et al. *JAMA Network Open.*

Q10 Do you believe that *USMLE STEP 2CK* scores accurately predict a resident's ability to perform clinically in your specialty?

- ◯ Yes (1)
- ◯ Neutral (2)
- ◯ No (3)

Q11 Will a student's medical school rank be considered more after *USMLE STEP 1* becomes pass/fail?

- ◯ Yes (1)
- ◯ Neutral (2)
- ◯ No (3)

Q12 After *USMLE STEP 1* becomes pass/fail, do you believe students will be better prepared clinically?

- ◯ Yes (1)
- ◯ Neutral (2)
- ◯ No (3)

© 2022 Patel OU et al. *JAMA Network Open.*

Q13 Please rank the following factors for a residency application, with 1 being the factor of most importance (***prior*** to STEP 1 going P/F)
______ USMLE STEP 1 Score (1)
______ Mean number of Research Experiences in Specialty (2)
______ Number of Abstracts, Presentations, and Publications (3)
______ Gold Humanism Honor Society (GHHS) membership (4)
______ Volunteer Experience (5)
______ Alpha Omega Alpha (AOA) membership (6)
______ Clerkship Grades (7)
______ Dean's Letter (8)
______ Personal Statement (9)
______ Preclinical Grades (10)
______ Letters of Recommendation in the Specialty (11)
______ Class Rank/Quartile (12)
______ Away rotation in your specialty at another institution (13)
______ Applicant has Graduate Degree (PhD,MPH, MBA,etc.) (14)
______ Involvement and Leadership (15)
______ USMLE STEP 2 CK Score (16)
______ Graduated from one of the 40 U.S. medical schools with the highest NIH funding (18)

Q14 Please rank the following factors for a residency application, with 1 being the factor of most importance (***after*** STEP 1 going P/F)
______ Mean number of Research Experiences in Specialty (2)
______ Number of Abstracts, Presentations, and Publications (3)
______ Gold Humanism Honor Society (GHHS) Member (4)
______ Volunteer Experience (5)
______ Alpha Omega Alpha (AOA) Member (6)
______ Clerkship Grades (7)
______ Dean's Letter (8)
______ Personal Statement (9)
______ Preclinical Grades (10)
______ Letters of Recommendation in the Specialty (11)
______ Class Rank/Quartile (12)
______ Away rotation in your specialty at another institution (13)
______ Applicant has Graduate Degree (PhD,MPH, MBA,etc.) (14)
______ Involvement and Leadership (15)
______ USMLE STEP 2 CK Score (16)
______ Graduated from one of the 40 U.S. medical schools with the highest NIH funding (18)

© 2022 Patel OU et al. *JAMA Network Open.*
